# Andrographolide Protects PC12 Cells Against β-Amyloid-Induced Autophagy-Associated Cell Death Through Activation of the Nrf2-Mediated p62 Signaling Pathway

**DOI:** 10.3390/ijms19092844

**Published:** 2018-09-19

**Authors:** Lili Gu, Qingqing Yu, Qin Li, Lingxi Zhang, Hong Lu, Xinyue Zhang

**Affiliations:** 1 Institute of Materia Medica, Zhejiang Academy of Medical Sciences, Hangzhou 310013, China; 17826865042@163.com (L.G.); liqin@zjams.com.cn (Q.L.); m18980211993@163.com (L.Z.); 2 Zhejiang Chinese Medical University, College of Pharmaceutical science, Hangzhou 310053, China; m15757196155_2@163.com (Q.Y.); luhong@zcmu.edu.cn (H.L.)

**Keywords:** andrographolide, β-amyloid, Nrf2, p62, autophagy, Alzheimer’s disease

## Abstract

Recent studies mentioned that Andrographolide (Andro), the main bioactive component of traditional Chinese medicine *Andrographis paniculata*, may be a potential natural product for treating Alzheimer's disease, but the underlining mechanism remains to be discovered. In this study, we investigated whether Andro regulates the nuclear factor E2-related factor 2 (Nrf2)/Sequestosome 1 (p62) signaling pathway and activates autophagy to protect neuronal PC12 cells from the toxicity of the β-amyloid (Aβ) peptide. Our results revealed that Andro protected and rescued PC12 cells from Aβ_1–42_-induced cell death and restored abnormal changes in nuclear morphology, lactate dehydrogenase, malondialdehyde, intracellular reactive oxygen species, and mitochondrial membrane potential. RT-PCR and Western blotting analysis demonstrated that Andro activated autophagy-related genes and proteins (Beclin-1 and LC3); meanwhile, it also augmented the Nrf2 and p62 expression in mRNA and protein levels, and reduced p-tau and p21 protein expression in Aβ_1–42_-stimulated cells. Then, further study showed that the pre-transfection of cells with Nrf2 small interfering RNA (siRNA) resulted in the downregulation of p62, Beclin-1, and LC3 proteins expression, as well as the upregulation of p21. Furthermore, the pre-transfection of cells with p62 siRNA didn’t block the Nrf2 protein expression, accompanying with an elevated p21. Taken together, these results showed that Andro significantly ameliorated cell death due to Aβ_1–42_ insult through the activation of autophagy and the Nrf2-mediated p62 signaling pathway.

## 1. Introduction

**Figure 1:**
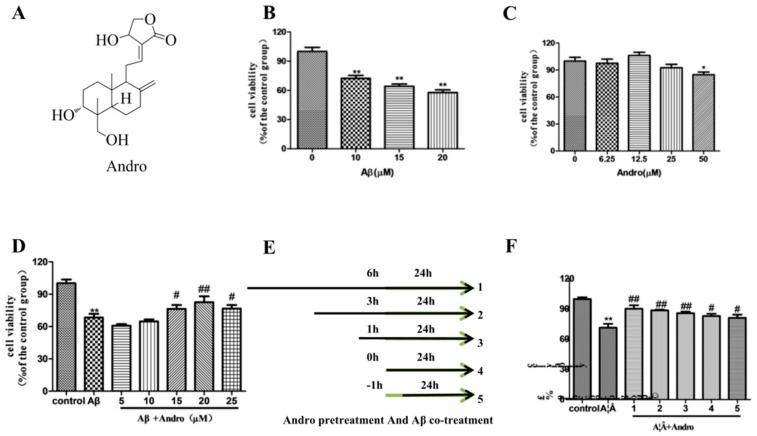
Andrographolide (Andro) protected PC12 cells from amyloid beta (Aβ)_1–42_ neurotoxicity**.** (**A**) The chemical structure of Andro. Cells were cultured with different concentration of Aβ_1–42_ (0, 10, 15, and 20 µM); (**B**) or Andro (6.25, 12.5, 25, and 50 µM); (**C**) for 24 h, respectively; (**D**) Cells were co-treated with 10 µM of Aβ_1–42_ and Andro (5, 10, 15, 20, and 25 µM) for 24 h; (**E**,**F**) Cells were pre-treated with Andro (20 μM) for 6, 3, 1, and 0 h and then incubated with 10 μM of Aβ_1–42_ for a further 24 h, cells were incubated with 10 μM of Aβ_1–42_ for 1 h, and then co-treated with Andro (20 μM) for 24 h, and the cell ability was detected by methyl thiazolyl tetrazolium (MTT) assay (*n* = 3). * *p* < 0.05, ** *p* < 0.01, versus the control; ^#^ *p* < 0.05, ^##^ *p* < 0.01 versus Aβ_1–42_ group was considered statistically significant differences.

Andrographolide (Andro), which is a lactone diterpenoid (Figure 1A), is present in the medicinal plant *Andrographis paniculata*, and is well-known for showing a variety of pharmacological actions including anti-inflammatory [1], anticancer [2] and immunomodulatory activities [3]. It has been widely used in clinics for the treatment of fever, cold, inflammation, diarrhea, and other infectious diseases. Andro is an apolar compound with low molecular weight that easily passes through the blood–brain barrier [4]. There is growing awareness in Andro as an efficient molecule with a potential property for the treatment of central nervous system diseases. Of note, recent studies have shown that Andro could reduce cognitive impairment in models of Alzheimer’s disease (AD), such as a rat model of streptozotocin intracerebroventricularly injection [5], AbetaPPswe/PS-1 transgenic mice [6], or a natural model of AD (Octodon degus) [7]. More and more researchers have begun to pay attention to explore its underlining mechanism on the treatment of AD [8,9,10].

AD is the most common neurodegenerative disease and a leading cause of dementia in the elderly. The confirmatory AD diagnosis is based on two major histopathologic hallmarks: senile plaques, which are extracellular deposits of amyloid beta (Aβ) peptides, and intraneuronal neurofibrillary tangles, which are somatic inclusions of hyperphosphorylated microtubule- associated protein tau. Autophagy is characterized as an essential cellular self-renewal process that promotes the degradation of damage components and the recycling of building blocks to maintain energy homeostasis and facilitate cell survival under stress. It has been reported that autophagy plays an important role in the generation and metabolism of Aβ, as well as the assembling of tau, and thus its malfunction may lead to the progress of AD [11]. The accumulation of Aβ and the consequent AD phenotype were accompanied by the downregulation of autophagy-related gene expression [12]. However, the underlying mechanism whereby Andro regulates autophagy remains largely unknown. The dual regulatory effects of Andro on autophagy have been reported in previous studies, with both inhibitory [13,14] and stimulatory roles for Andro in autophagy [15,16]. Therefore, there is a considerable need to explore whether the activation of autophagy is involved in the process of Andro for AD treatment.

Aβ induces neuronal apoptosis by targeting mitochondria, including the promotion of mitochondrial fission, the disruption of mitochondrial membrane potential (MMP), and increasing intracellular reactive oxygen species (ROS) level [17]. Furthermore, autophagy inhibited ROS generation by facilitating mitochondrial turnover [18]. Meanwhile, the accumulation of too-high levels of ROS is dangerous and defined as oxidative stress. Nuclear factor E2-related factor 2 (Nrf2) plays a vital role in protecting cells against oxidants. There is also increasing evidence supporting endogenous antioxidant defense enhancement by Andro through Nrf2 activation [1], and the Nrf2 pathway is also a potential therapeutic target in neurodegenerative disease [19]. On the other hand, autophagy alteration triggered the Nrf2 signaling pathway with consequences such that the autophagy inducer causes the Nrf2 protein gathered as a negative feedback loop [20]. Previously, the high expression of sequestosome 1 (p62), which is a major cargo receptor for selective autophagy, could competitively interact with Keap1 (kelch-like ECH-associated protein 1), the inhibitor of Nrf2, leading to the constitutive activation of Nrf2. Nrf2 also upregulates p62, and has a positive feedback by binding directly to the ARE site of p62. It is interesting to note that p62 is present in neurofibrillary tangles, and p62 transcription seems to be decreased in AD, leading to diminished p62 synthesis [21]. Therefore, our study would the first time to explore Andro activate autophagy to protect neuronal cells against Aβ-related neurotoxicity, and then to further assess the role of the Nrf2/p62 pathway in Aβ-stimulated PC12 Cells.

## 2. Results

### 2.1. Andro Protected PC12 Cells from Aβ_1–42_ Neurotoxicity

Aβ-induced apoptosis in PC12 cells was a common and reliable cellular toxicity model for AD related studies in vitro. We first tested the cytotoxicity of the most usual used peptide Aβ_1–42_ on PC12 cells by MTT assay in our laboratory conditions. As shown in Figure 1B, the exposure of cells to different concentrations of Aβ_1–42_ for 24 h resulted in a notable decrease of the cell viability in a concentration-dependent manner. Compared with that in the control group, the cell viability in the 10 µM Aβ_1–42_ group was about 70% (*p* < 0.01). To evaluate the protective effects of Andro, the result (Figure 1C) revealed that the treatment of less than 50 μM Andro didn’t result in dominant cell death. Then, co-treated with 10 µM Aβ_1–42_ and Andro (5–25 µM) for 24 h, 20 µM of Andro significantly attenuated Aβ_1–42_-induced cell death (*p* < 0.01) (Figure 1D). In addition, compared with the Aβ_1–42_ injury group, the cell viability was rescued by pre-treatment with 20 µM Andro for 6, 3, and 1 h (*p* < 0.01) (Figure 1E,F). Thus, pre-treatment with 20 µM of Andro for 1 h and then incubation with 10 μM of Aβ_1–42_ was determined to be the optimal condition for the following experiment.

**Figure 2:**
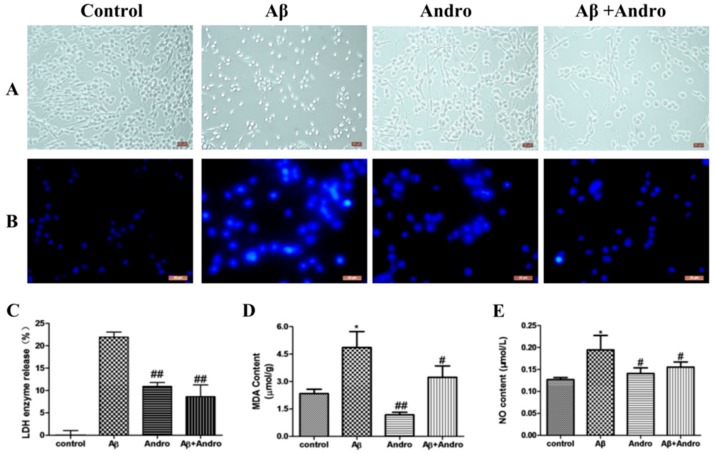
Andro reduced the morphology damage, lactate dehydrogenase (LDH) release, MDA and NO levels in Aβ_1–42_-treated PC12 cells. Cells were pre-treatment with or without 20 µM of Andro for 1 h before exposed to 10 μM of Aβ_1–42_ for 24 h. Then, the cellular morphology was observed and photographed by inverted light microscopy with 10× magnification (**A**); Cells was detected by staining with Hoechst 33258 and visualized by fluorescence microscopy with 20 × magnification; (**B**). The release of LDH (**C**), the levels of MDA (**D**), and NO (**E**) were examined by using the reagent kits. * *p* < 0.05 versus the control; ^#^ *p* < 0.05, ^##^ *p* < 0.01 versus the Aβ_1–42_ group was considered statistically significant differences.

Morphological damage and nuclei condensation was observed in PC12 cells after exposure to Aβ_1–42_ for 24 h in Figure 2A,B. Pre-treatment with Andro definitely improved these changes.

### 2.2. Andro Attenuated the Productions of LDH, MDA, and NO in Aβ_1–42_-Stimulated PC12 Cells

The protective activity of Andro was also confirmed by the lactate dehydrogenase (LDH) assay. The content of LDH that was released into the extracellular medium from cells without any treatment was considered as 0%. As shown in Figure 2C, pre-treated cells with Andro significantly reduced LDH leakage in the supernatant of Aβ_1–42_-injured cells (*p* < 0.01). Simultaneously, intracellular malonaldehyde (MDA) concentrations and nitric oxide (NO) levels were significantly increased in Aβ_1–42_-treated cells compared with the control group cells (*p* < 0.05), and Andro decreased intracellular MDA concentrations and NO levels in Aβ_1–42_-induced cells_,_ (*p* < 0.05) (Figure 2D,E), which indicated that Andro could protect cells against oxidative damage from Aβ_1–42_.

### 2.3. Andro Reduced the Levels of ROS, MMP, Cytc, and Bax in Aβ_1–42_-Stimulated PC12 Cells

**Figure 3:**
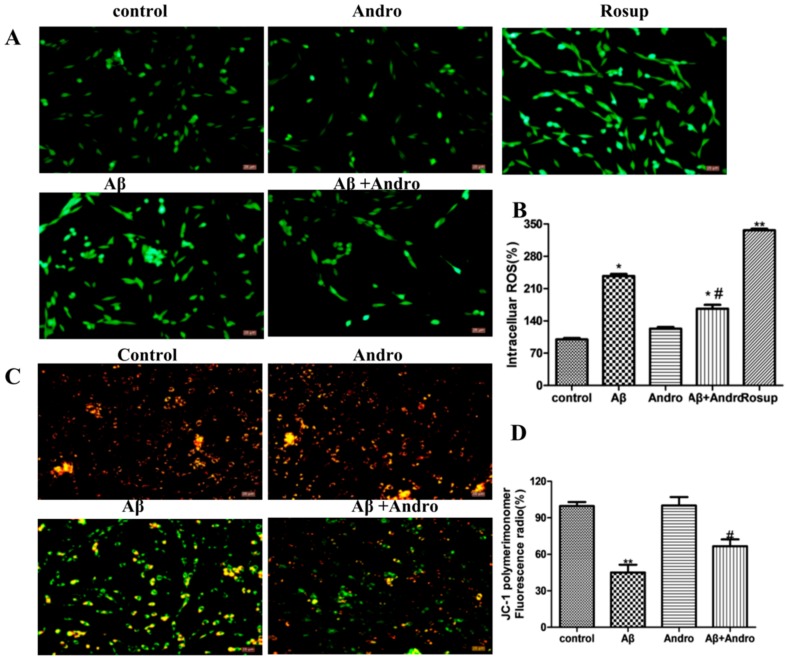
Andro attenuated Aβ_1–42_-induced reactive oxygen species (ROS) production and mitochondrial membrane potential (MMP) loss in PC12 cells. After pre-treatment with 20 μM of Andro for 1 h, PC12 cells were incubated with 10 μM Aβ_1–42_ for another 24 h. (**A**,**B**) Intracellular ROS level was determined by the fluorescent dye 2,7-dichlorofluorescein-diacetate (DCFH-DA) Reagent and visualized by fluorescence microscopy with 20× magnification, and Rosup was used as a positive control group; (**C**,**D**) MMP was determined by the JC-1 assay and visualized by fluorescence microscopy with 20× magnification. * *p* < 0.05, ** *p* < 0.01 versus the control; ^#^ *p* < 0.05 versus the Aβ_1–42_ group, were considered statistically significant differences.

**Figure 4:**
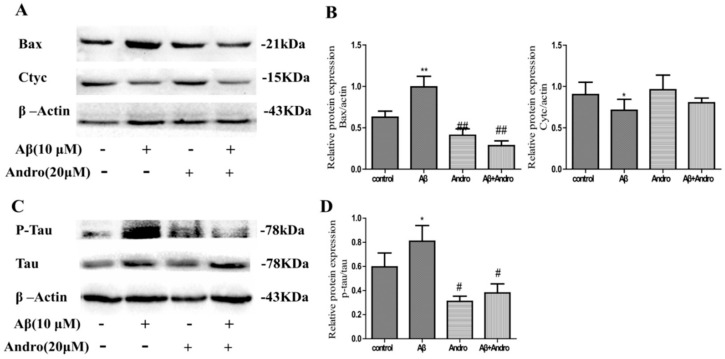
Andro alters Aβ_1–42_-induced cytochrome C (Cytc), Bax, and p-Tau/Tau protein expression levels in PC12 cells. After pre-treatment with 20 μM of Andro for 1 h, PC12 cells were incubated with 10 μM of Aβ_1–42_ for another 24 h. (**A**,**C**) The expression of Cytc, Bax, p-tau, and tau protein levels was determined by Western blotting with specific antibodies; (**B**,**D**) Quantification of Cytc, Bax, p-tau, and tau protein expression levels. * *p* < 0.05, ** *p* < 0.01 versus the control; ^#^ *p*< 0.05, ^##^ *p* < 0.01 versus the Aβ_1–42_ group were considered statistically significant differences.

Previous studies reported that the toxicity of Aβ_1–42_ was mediated through the generation of ROS, and mitochondrial inhibition leads to the loss of MMP involved in the progression of AD and neuron apoptosis [17]. Here, we investigated whether Andro blocked Aβ_1–42_-induced oxidative stress in PC12 cells. As expected, Andro significantly reduced the intracellular production of ROS induced by Aβ_1–42_ (*p* < 0.05) (Figure 3A,B). The results (Figure 3C,D) revealed that Andro pre-treatment significantly prevented the declined of MMP induced by Aβ_1–42_ (*p* < 0.05). In addition, the excess ROS generally lead to apoptosis through changes in the expression of the bcl-2 family and apoptosis-related proteins; thus, the alterations of the cytochrome C (Cytc) and rabbit bcl-2 related X protein (Bax) protein were measured. As shown in Figure 4A,B, Aβ_1–42_ decreased Cytc expression, and increased pro-apoptotic protein Bax levels in PC12 cells (*p*< 0.05), while pre-treatment with Andro could block the increase of pro-apoptotic protein Bax levels (*p* < 0.05) and reduce cell apoptosis to some extent.

### 2.4. Andro Lowered the Protein Levels of Phospho-Tau/Tau in Aβ_1–42_-Stimulated PC12 Cells

Given that the pathological phenotypes of Aβ deposition and the phosphorylation of tau are hallmarks in AD, in order to confirm the Andro-interfered impairment of tau hyperphosphorylation in Aβ_1–42_-stimulated PC12 cells, the levels of phospho-tau and tau were checked. As illustrated in Figure 4C,D, Aβ_1–42_ led to an increase in the ratio of Phospho-tau/tau protein levels in PC12 cells (*p* < 0.05), which is an effect abolished obviously by Andro (*p* < 0.05).

### 2.5. Andro Altered the mRNA and Protein Levels of Autophagy Markers in Aβ_1–42_-Stimulated PC12 Cells

**Figure 5:**
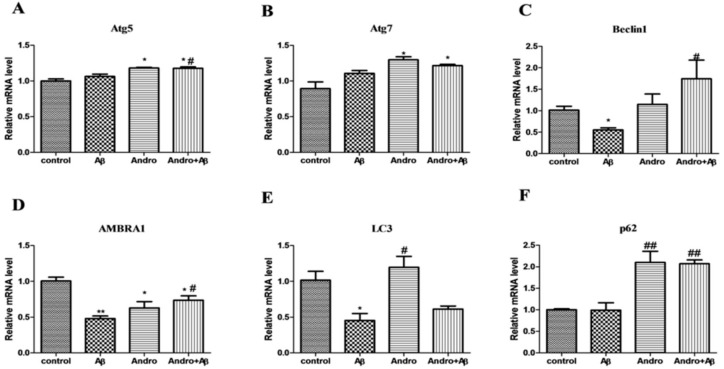
Effect of Andro on Aβ_1–42_-induced autophagy-related mRNA expression levels in PC12 cells. After pre-treatment with 20 μM of Andro for 1 h, cells were incubated with 10 μM of Aβ_1–42_ for another 24 h, and real-time qPCR analysis of the mRNA levels of: autophagy-related gene (*Atg*) 5 (**A**); *Atg 7* (**B**); *Beclin-1* (**C**); *AMBRA1* (**D**); *LC3* (**E**); and *p62* (**F**). * *p* < 0.05, ** *p* < 0.01 versus the control; ^#^ *p* < 0.05, ^##^ *p* < 0.01 versus the Aβ_1–42_ group were considered statistically significant differences.

Autophagy plays a key role in neuronal function being responsible for the removal of damaged proteins and organelles. Autophagosomes are already demonstrated to be an active compartment for Aβ generation. Autophagy-related genes (*Atg*) are involved in the control of autophagosome formation. Aβ_1–42_ had no obvious effect in both *Atg 5* and *Atg 7* mRNA levels, but Andro strengthened Atg 5 (*p* < 0.05) and *Atg 7* mRNA levels in Aβ_1–42_-stimulated PC12 cells (Figure 5A,B). The decrease in mRNA levels of *Beclin-1* and autophagy and Beclin-1 regulator 1 (*AMBRA1*), were observed in Aβ_1–42_-induced PC12 cells (*p* < 0.05, *p* < 0.01) (Figure 5C,D), and Andro prevented Aβ-induced *Beclin-1* and *AMBRA1* mRNA levels’ downregulation (*p* < 0.05), suggesting a disruption in the elongation phase of the autophagosome formation. *LC3* and *p62* are indicators of autophagy. The *LC3* mRNA level was downregulated in an Aβ-induced group (*p* < 0.05), and upregulated in an Andro-induced group, while co-treatment Aβ and Andro induced a slight increase compared to the Aβ group (Figure 5E). As shown in Figure 5F, there was no significant alterations in *p62* mRNA levels between the control and the Aβ group, while Andro led to an evident increase in Aβ_1–42_-stimulated cells (*p* < 0.01). These results hinted that autophagy was restrained after Aβ intervened, and Andro maybe reversed this effect and promoted the expression of autophagy-related genes, particularly in p62 mRNA.

**Figure 6:**
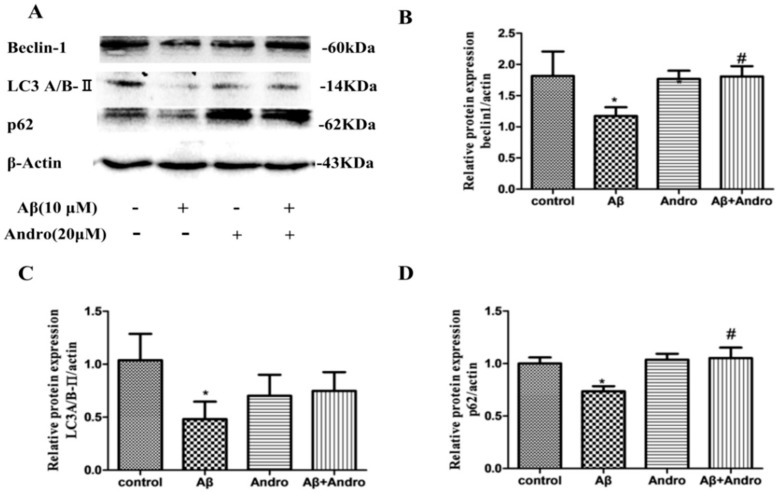
Effect of Andro on Aβ_1–42_-induced Beclin-1, LC3A/B, and p62 protein expression levels in PC12 cells. After pre-treatment with 20 μM of Andro for 1 h, cells were incubated with 10 μM of Aβ_1–42_ for another 24 h. The expression of proteins levels was determined by Western blotting with specific antibodies (**A**). Quantification of Beclin-1 (**B**), LC3A/B (**C**), and p62 (**D**) protein expression levels. * *p* < 0.05 versus the control; ^#^ *p* < 0.05 versus Aβ_1–42_ group was considered statistically significant differences.

To further confirm that Andro-induced enhancement of autophagic activities, we investigated its markers Beclin-1, p62 and LC3A/B-II protein levels. As shown in Figure 6A, Aβ_1–42_ induced the downregulation of Beclin-1, LC3A/B-II, and p62 protein levels *(p* < 0.05). Andro increased LC3A/BII protein levels in Aβ_1–42_-treated PC12 cells (Figure 6C). Compared to the Aβ_1–42_-treated group, a remarkable increase in Beclin-1 and p62 protein levels was observed in the Aβ and Andro co-treated group *(p* < 0.05) (Figure 6B,D), which was in consistent with the mRNA results, suggesting that Andro promoted autophagy, and p62 signaling may play a crucial role in the protection of Aβ-induced cell damage.

### 2.6. P62 Induced by Andro Played an Important Role in Preventing Cell Toxicity in Aβ_1–42_-Stimulated PC12 Cells via Autophagy

**Figure 7:**
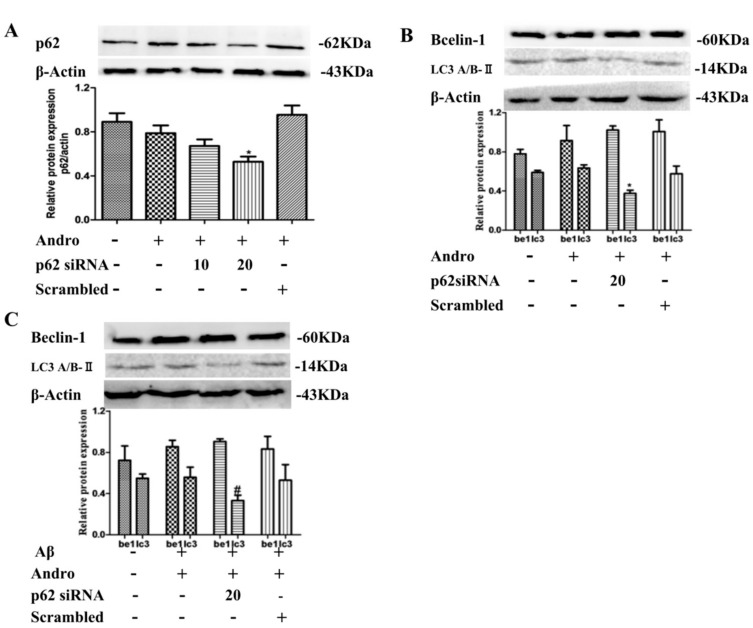
Effect of p62 small interfering RNA (siRNA) on Beclin 1 and LC3A/B-II protein expression levels in PC12 cells. (**A**,**B**) PC12 cells were transfected either with p62 siRNA (10 or 20 µM) or scrambled siRNA. Cells were then treated with Andro (20 µM) for 24 h, and p62, Beclin 1, and LC3A/B-II protein expression levels were evaluated by Western blot analysis; (**C**) After transfection with 20 µM of p62 siRNA, cells were then pre-treated with Andro (20 µM) for 1 h, followed by stimulation with Aβ (10 µM) for an additional 24 h. Then, Beclin 1 and LC3A/BII protein expression levels were evaluated by Western blot analysis. * *p* < 0.05 versus the blank control; ^#^ *p* < 0.05 versus the Andro + Aβ_1–42_ group were considered statistically significant differences.

P62 is a receptor that facilitates selective autophagy by interacting simultaneously with cargoes and LC3 protein on the autophagosome to maintain cellular homeostasis. Next, we examined the functional role of p62 in autophagy by Andro. Cells were transfected with small interfering RNAs (siRNA) targeting p62. The transfection efficiency of p62 siRNA was monitored by Western blot analysis. As shown in Figure 7A, 20 μM of p62 siRNA had a better inhibition effect on p62 protein expression in Andro-treated cells (*p* < 0.05). The knockdown of p62 reduced LC3A/B-II protein expression (*p* < 0.05), and didn’t change Beclin-1 in Andro-treated cells (Figure 7B), which indicated that Andro-induced autophagy may be weakened in p62 siRNA-treated cells. Then, in PC12 cells co-treated with Andro and Aβ_1–42_, p62 knockdown led to a decrease in protein levels of LC3A/B-II (*p* < 0.05), and had no obvious change on Beclin-1 protein levels (Figure 7C). Therefore, the upregulation of p62 induced by Andro participated to some extent in autophagy form in Aβ_1–42_-stimulated PC12 cells.

### 2.7. Andro Enhanced the Nrf2 Activation and Weakened the p21 Expression in Aβ_1–42_-Stimulated PC12 Cells

**Figure 8:**
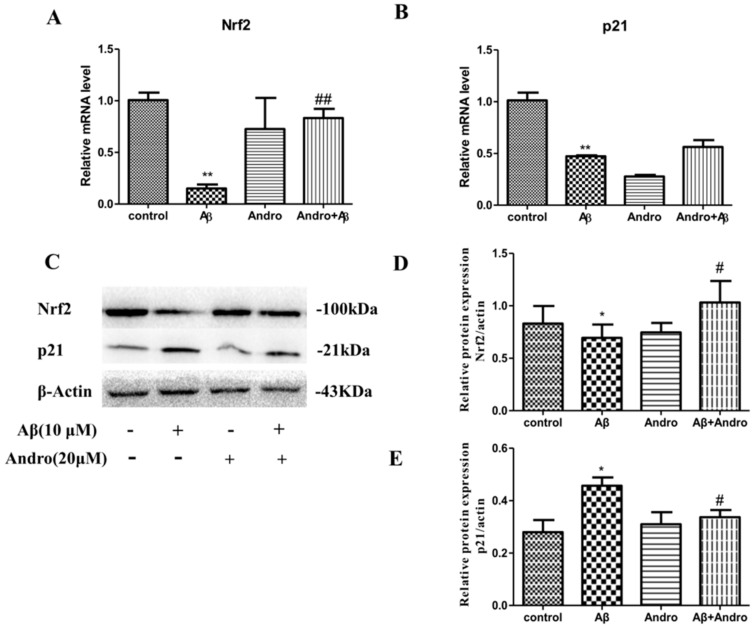
Effect of Andro on Aβ_1–42_-induced *p21* and *Nrf2* mRNA and protein expression levels in PC12 cells. After pre-treatment with 20 μM of Andro for 1 h, PC12 cells were incubated with 10 μM of Aβ_1–42_ for another 24 h. Real-time qPCR analysis of the mRNA levels of *p21* (**A**) and *Nrf2* (**B**) in cells, and (**C**) Western blotting analysis of the protein levels of p62, Nrf2, and β-Actin in cells. Quantification of Nrf2 (**D**) and p21 (**E**) protein expression levels. * *p* < 0.05, ** *p* < 0.01 versus the control; ^#^ *p* < 0.05, ^##^ *p* < 0.01 versus the Aβ_1–42_ group were considered statistically significant differences.

Nrf2 is implicated in mitigating oxidative stress and inflammation, which are both potentially key components in AD pathogenesis. When we treated cells with Aβ_1–42_, the expression of *p21*, a cell cycle inhibitor, in mRNA levels (Figure 8A) was decreased (*p* < 0.01), but there was an increase in protein levels (*p* < 0.05) (Figure 8C,D), suggesting that cell proliferation was inhibited. The expression of *Nrf2* in mRNA and protein levels (Figure 8B,E) was decreased in Aβ_1–42_-treated PC12 cells (*p* < 0.01, *p* < 0.05). Andro obviously decreased the p21 protein levels (*p* < 0.05) and increased the *Nrf2* mRNA and protein levels in Aβ_1–42_-stimulated PC12 cells (*p* < 0.01, *p* < 0.05), which hinted that Nrf2 activation may be involved in the neuroprotective property of Andro.

### 2.8. Nrf2 Signaling Pathway Mediated by Andro Was Involved in p62 Expression and Autophagy Induction in Aβ_1–42_-Stimulated PC12 Cells

**Figure 9:**
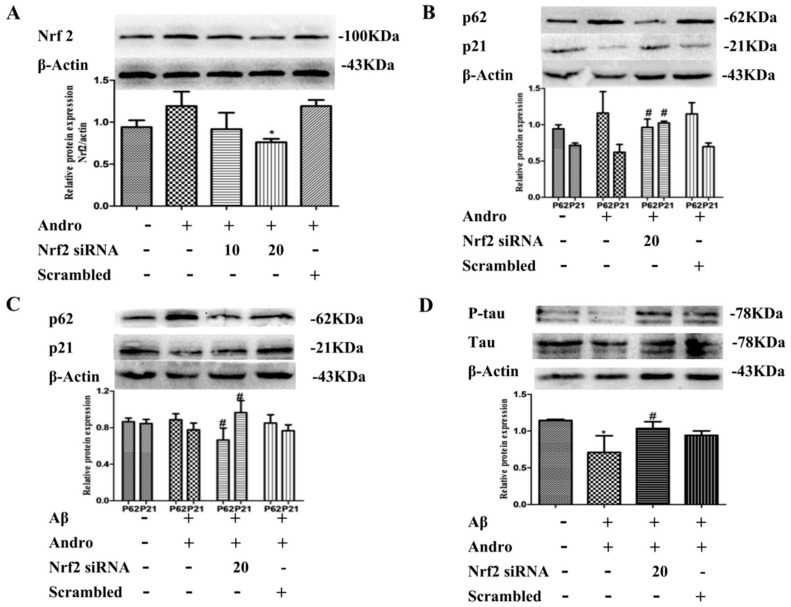
Effect of Nrf2 siRNA on p62, p21, and p-tau/tau protein expression levels in PC12 cells. (**A**,**B**) Cells were transfected either with Nrf2 siRNA (10 or 20 µM) or scrambled siRNA, and then treated with Andro (20 µM) for 24 h. Then, Nrf2, p62, and p21 protein levels were evaluated by Western blot analysis; (**C**,**D**) After transfection with 20 µM of Nrf2 siRNA, cells were then pre-treated with Andro (20 µM) for 1 h followed by stimulation with Aβ (10 µM) for an additional 24 h. Then, p62, p21, and p-tau/tau protein expression levels were evaluated by Western blot analysis. * *p* < 0.05 versus the blank control; ^#^ *p* < 0.05 versus Andro+Aβ_1–42_ group were considered statistically significant differences.

It is reported that Andro has an impact on the Nrf2 signaling pathway, and Nrf2 can also regulate changes in autophagy directly and indirectly. We examined the functional role of Nrf2 in Aβ_1–42_-stimulated PC12 cells. Cells were transfected with siRNA targeting Nrf2. As indicated in Figure 9A, 20 μM of Nrf2 siRNA had a better effect in Andro-treated cells. Next, we found that Nrf2 knockdown significantly downregulated p62 and upregulated p21 protein expression in Andro-treated cells (*p* < 0.05) (Figure 9B), implying a crucial role for Nrf2 signaling in p62 expression. In Andro and Aβ_1–42_ co-stimulated PC12 cells, Nrf2 knockdown also led to a decrease in p62, and an increase in p21 in protein levels (*p* < 0.05) (Figure 9C). Meanwhile, Nrf2 knockdown significantly increased p-tau/tau protein levels (*p* < 0.05) (Figure 9D), which meant that the Nrf2 signaling pathway mediated by Andro could reduce the production of p-tau protein and protect cells from Aβ_1–42_ toxicity.

**Figure 10:**
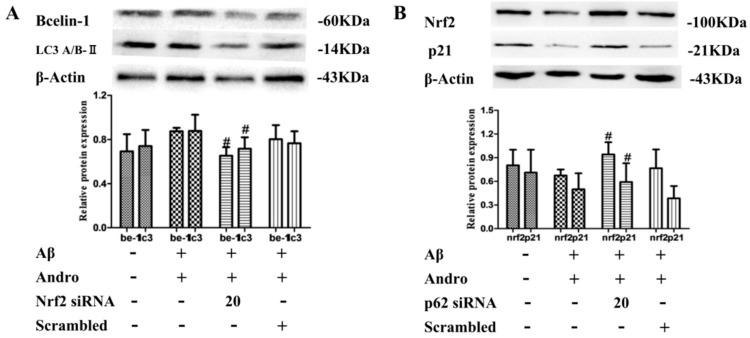
Nrf2/p62 signaling pathway mediated by Andro involved autophagy induction in Aβ_1–42_-stimulated PC12 cells. After transfection with 20 µM of Nrf2 siRNA (**A**) or p62 siRNA (**B**), cells were then pre-treated with Andro (20 µM) for 1 h followed by stimulation with Aβ (10 µM) for an additional 24 h, Beclin-1, LC3A/BII, Nrf2, and p21 protein expression levels were evaluated by Western blot analysis. ^#^ *p* < 0.05 versus the Andro + Aβ_1–42_ group were considered statistically significant differences.

Furthermore, in Andro and Aβ_1–42_ co-stimulated PC12 cells, Nrf2 knockdown significantly inhibited Andro-induced Beclin-1 and LC3A/BII accumulation (*p* < 0.05) in protein levels (Figure 10A), which was the opposite of the results obtained from p62 knockdown (Figure 8C), indicating the vital role of Nrf2 signaling in Andro-induced autophagy activation. In addition, compared with the Andro and Aβ_1–42_ co-stimulated group, the knockdown of p62 increased Nrf2 and p21 expressions in protein levels (*p* < 0.05) (Figure 10B). Taken together, these results seemed to imply that the transcriptional activation of Nrf2 mediated by Andro was involved in p62 protein accumulation, and promoted autophagy in Aβ_1–42_-induced PC12 cells.

## 3. Discussion

Andro is a natural diterpenoid with multiple bioactivities. It was reported a few years ago that Andro, as an anti-inflammatory drug, effectively ameliorated astrocytic pro-inflammatory reactions and prevented PC12 cells from H_2_O_2_-induced death at the doses of 5, 10, and 50 µM, which may make it a candidate for the treatment of neurodegeneration. This has generated a considerable amount of interest [22]. In our experiment, we conducted research to find out the possible protective effect and mechanism of Andro on PC12 cells for Aβ_1–42_-induced death. Firstly, the 10-µM Aβ_1–42_-induced cell model was established, and it was found that pre-treatment with 20 µM of Andro could effectively improve cell viability and reduce the change of cell morphology, LDH release, and NO levels under our experimental condition. By the way, Andro regulated the ROS levels, MDA content, and MMP as well as Cytc and Bax expression. The contents of MDA and ROS are indicators of oxidative stress. The upregulation of pro-apoptotic protein Bax can be translocated to the mitochondrial membrane, which releases Cytc, changes MMP, and causes mitochondrial cell death. It was showed that Andro weakens Aβ_1–42_-induced intracellular oxidative stress and apoptosis.

Autophagy may act either as pro-survival or pro-death. Andro enhanced autophagy in many cancer cells, including human osteosarcoma cells [15], HeLa cells [23], and human liver cancer cells [24]. During autophagy progression, Atg 5, Atg 7, and Beclin-1 play an essential role in the formation of autophagy. Furthermore, LC3A/B is cleaved to a lapidated form, LC3 A/B-II, which localizes and aggregates onto the membranes of autophagosomes, demonstrating the condition of autophagy. P62 is a multidomain protein that interacts with the autophagy machinery as a key adaptor of target cargo, which cross-link LC3-decorated autophagosomal membranes with ubiquitinated targets destined to degradation [25]. In this study, our results showed that Andro altered the mRNA of *Atg 5, Atg 7, Beclin-1*, *AMBRA1*, *p62,* and *LC3A/B*, and upregulated the protein levels of Beclin-1, p62, and LC3A/B-II in Aβ_1–42_-stimulated PC12 cells, suggesting that the protective mechanism of Andro was related to autophagy activation, and the change of p62 protein was the most notable. Thus, we explored the role of p62 in Andro-induced autophagy activation. The results showed that the knockdown of *p62* decreased the LC3A/B-II expression in Aβ and Andro co-treated cells, which hinted that p62 had a place in Andro-induced autophagy activation.

Evidence from the literature showed that Andro activates transcription factor Nrf2-mediated HO-1 protein expression and inhibits A𝛽42-overexpressed microglial BV-2 cell activation [9]. Nrf2-induced compounds also have shown therapeutic efficacy in many neurodegenerative disease models [26]. The abnormal hyperphosphorylation of tau is a hallmark of AD. In Aβ_1–42_-stimulated PC12 cells, we observed the same phenomenon: that *Nrf2* expression increased in mRNA and protein levels, and meanwhile, the expression of p-tau protein decreased with Andro treatment. There is also evidence that Nrf2 reduces levels of p-tau protein by inducing autophagy adaptor protein NDP52 [27]. Therefore, we next explored the role of Nrf2 on the regulation of autophagy as well as p-tau. The results showed that *Nrf2* knockdown lowered the LC3A/B-II and Beclin-1 protein expression induced by Andro in Aβ-treated cells, and raised the p-tau protein expression, hinting that inhibition of Nrf2 activation would downregulate the autophagy and reinforce cell injury.

Moreover, the Nrf2 antioxidant pathway can be regulated by p62 [21]. In normal condition, Keap1 binds with Nrf2 and recruits ubiquitin-proteasome factors, resulting in the ubiquitination and degradation of Nrf2. Interestingly, p62 is translocated to autophagosomes, leading to Ser351 phosphorylation in the Keap1-interacting region (KIR) motif, enhancing the interaction between p62-Keap1 and consequently Nrf2 translocation [21,28]. Nrf2 and p62 form positive feedback loops in PC12h cells [29]. In this study, we found that *Nrf2* knockdown decreased the p62 expression, but *p62* knockdown didn’t inhibit Nrf2 expression, and *Nrf2* or *p62* knockdown increased cell cycle inhibitor p21 protein expression in Aβ and Andro co-treated cells. There is evidence that p21 induction also results in the disruption of the interaction between Keap1 and Nrf2, implying a functional role for p21 in modulating Nrf2 activation [30]. In addition to its typical inhibitory role in cell cycle progression, p21 has been recently shown to regulate other biological functions, including the onset of cellular senescence, autophagy, and inflammation. So the p21/Nrf2 pathway may act as the upstream signaling of p62 expression in the course of protecting cells from Aβ_1–42_ toxicity by Andro, andfurther experiments were needed to properly illuminate this pathway.

In conclusion, our results have shown that Andro can enhance cell ability and reduce the oxidative damage that occurs in Aβ_1–42_-induced PC12 cells. What’s more, Andro altered the expression levels of autophagy-related markers and induced autophagy. Meanwhile, it obviously increased Nrf2 and p62 expression. Nrf2 knockdown decreased the p62 expression, consequently downregulated the autophagy, and raised the p-tau levels. Evidence was presented for the first time that Andro protected PC12 cells from Aβ_1–42_-induced cell death through the activation of the Nrf2-mediated p62 signaling pathway.

## 4. Materials and Methods

### 4.1. Chemicals and Reagents

Andro presents as a white powder; its purity is 98%, and it was obtained from Sigma-Aldrich (St. Louis, MO, USA) (lot number: MKCD1369), which was dissolved in dimethyl sulfoxide (DMSO, Sigma Shanghai, China) and then diluted in the culture medium. The highest DMSO concentration of medium (0.5%) did not have a significant effect on the determined cellular functions. Aβ_1–42_ (P9001) was purchased from the Beyotime Institute of Biotechnology (Shanghai, China).

Highly differentiated PC12 cells were purchased from the Cell Center of the Chinese Academy of Medical Sciences (Beijing, China). Cells were cultured in Dulbecco’s modified Eagle’s medium (DMEM, Gibco, Gaithersburg, MD, USA), which was supplemented with 4.5 g/L d-glucose and 10% fetal bovine serum (FBS) and incubated at 37 °C with 5% CO_2_ humidified atmosphere.

### 4.2. MTT Assay

Methyl thiazolyl tetrazolium (MTT) assay was used to detect the cell survival rate. Briefly, cells were seeded into a 96-well plate at a density of 2 × 10^4^ cells/mL. Medium containing a certain concentration of Andro or Aβ was added into each well in a volume of 100 μL at the appointed time. Then, 20-μL MTT solutions (Sigma, Shanghai, China) were added into each well, followed by incubation at 37 °C for 4 h. Then, 150 μL of DMSO was further added after discarding the culture medium. The crystals of formazan product were then dissolved by oscillating for 10 min. The optical density (OD) value was detected using a microplate reader (Bio-Rad, imark, Winooski, VT, USA) at a wavelength of 570 nm. The experiments were performed in triplicate.

### 4.3. Lactate Dehydrogenase (LDH) Release Assay

Cell cytotoxicity was measured by LDH released into the incubation medium when cellular membranes were destroyed. Cells were seeded into 96-well plates (5 × 10^3^ cells/well). After appropriate treatment, the activity of released LDH in the medium was determined according to the instructions of the LDH release assay kit (Beyotime, Shanghai, China). The OD value was detected using a microplate reader at a wavelength of 490 nm. All of the values of % LDH released were normalized to the control group.

### 4.4. Hoechst 33258 Staining

The apoptosis of cells was examined by staining with the DNA binding dye, Hoechst 33258. Cells were seeded in 12-well plates at a density of 2 × 10^5^ cell/mL. After appropriate treatment, the cell morphology was observed by invert/phase contrast microscopy (MOTIC AE2000, Beijing, China). Then, the culture medium were removed, and the cells were incubated with 0.5 mL of Hoechst 33258 (Beyotime, Shanghai, China) for 30 min at 37 °C. After being washed three times with PBS, the apoptotic cells were visualized using an inverted fluorescence microscope (Nikon TE2000, Tokyo, Japan).

### 4.5. Measurement of Intracellular ROS Levels

Intracellular ROS generation was assessed using an ROS assay kit (Beyotime, Shanghai, China). Cells were seeded in six-well plates at a density of 5 × 10^5^ cell/mL After appropriate treatment, the cells were incubated with DCFH-DA (10 µmol/L) in DMEM and incubated at 37 °C for 20 min. Rosup (volume ratio 1:1000) was used as a positive control group, and the fluorescence of DCFH was measured on inverted fluorescence microscope. The semiquantification of ROS levels was evaluated by using Image J software. All of the values of % ROS level were normalized to the control group.

### 4.6. MDA and NO Content Detection

Cells were seeded in six-well plates and collected after appropriate treatment. The protein concentration was determined using the bicinchoninic acid (BCA) protein assay kit with an absorption band of 570 nm (Beyotime, Shanghai, China). The formation of MDA—a substance produced during lipid peroxidation—was determined using a lipid peroxidation MDA assay kit (Beyotime, Shanghai, China).

Cells were seeded in 12-well plates, and 50 µL of cell culture medium was collected after appropriate treatment. NO levels were assessed by measuring the nitrite concentration of the cell culture medium with a total NO assay kit (Beyotime, Shanghai, China) according to the manufacturer’s instructions.

### 4.7. Measurement of Mitochondrial Membrane Potential (MMP)

A mitochondrial membrane potential (MMP) assay kit with JC-1 (Beyotime, Shanghai, China) was used to monitor the mitochondrial integrity. In brief, cells were seeded into black six-well plates (5 × 10^5^ cells/well). After appropriate treatment, the cells were incubated with JC-1 (10 μg/ml in medium) at 37 °C for 15 min, and then washed twice with PBS. The fluorescent signal in the cells was also observed and recorded with a fluorescent microscope. For signal quantification, the intensity of red fluorescence (excitation 560 nm, emission 595 nm) and green fluorescence (excitation 485 nm, emission 535 nm) were measured using a microplate reader. MMP was calculated as the ratio of JC-1 red/green fluorescence intensity, and the value was normalized to the control group.

### 4.8. Western Blotting

For Western blot analysis, cells were lysed using RIPA Lysis Buffer. The lysates were collected by scraping from the plates, and then they were centrifuged. Total protein samples were denatured and loaded on a SDS-polyacrylamide gel for electrophoresis, and then transferred onto PVDF transfer membranes (Millipore, Billerica, MA, USA). Membranes were blocked at room temperature for 2 h with blocking solution. Membranes were incubated overnight at 4 °C with primary antibodies: Bax (SC-493, Santa Cruz, Dallas, TX, USA), CytoC (11940S, CST, Beverly, MA, USA), β-action (4970s, CST, Beverly, MA, USA), tau (AF6141, Affinity, Cincinnati, OH, USA), phospho-tau (Ser396) (AF3148, Affinity, Cincinnati, OH, USA), Beclin-1 (#3495p, CST, Beverly, MA, USA), LC3A/B (AF5402, Affinity, Cincinnati, OH, USA), P62/SQSTM1 (AF5384, Affinity, Cincinnati, OH, USA), Nrf2 (ab62352, abcam, Cambridge, MA, USA) and p21 Waf1/Cip1 (12D1)Rabbit mAb (2947T, CST, Beverly, MA, USA) at a 1:1000 dilution in blocking solution. After three washings, the membranes were incubated with secondary horseradish peroxidase (HRP)-conjugated goat anti-rabbit immunoglobulin G. Then, the proteins were detected using an enhanced chemiluminescence detection kit. The images were obtained using a Mini-PROTEAN gel imaging system (Bio-rad, Hercules, CA, USA). The relative optical density of the digitized image was analyzed by Image J software.

### 4.9. Reverse Transcription Quantitative Polymerase Chain Reaction (RT-qPCR)

Total RNA was extracted from cells using the RNAprep pure cell/bacteria kit (Tiangen, Beijing, China). Reverse transcription of total RNA (50 ng–2 µg) was carried out in a final volume of 20 µL containing the following reagents (Tiangen, Beijing, China): 5× FastKing-RT SuperMix (4 µL) and RNase-free water. The reaction mixtures were then incubated at 42 °C (15 min) and 95 °C (3 min), and the cDNA samples were obtained. For PCR amplification, primers were designed based on the β-actin, p62, LC3, Atg 7, Atg5, Beclin-1, AMBRA1, Nrf2, and p21 sequences obtained from GenBank. β-actin: 5′-GCAGGAGTACGATGAGTCCG-3′ (forward) and 5′-ACGCAGCTCAGTAACAGTCC-3′ (reverse); p62: 5′-GTCAATTTCCTGAAGAATGTGGG-3′ (forward) and 5′-GAGTTCACCTGTG GATGGGTC-3′ (reverse); LC3: 5′-TTCTGGTCAGGTTCTCCCCA-3′ (forward) and 5′-CCCAGG ACTTGGTATGCTGG-3′ (reverse); Atg7: 5′-CCTGTCAGCCTGGCATTTGA-3′ (forward) and 5′-C AGACGGTCTCCTCGTCACT-3′ (reverse); Atg5:5′-CAGGACACCGAAGCATGACA-3′ (forward) and 5′-ATGGAATCTTCTGCCGCCTT-3′ (reverse); Beclin-1:5′-CTCGTCAAGGCGTCACTTCT -3′ (forward) and 5′-CCTCCATTCTTTAGGCCCCG-3′ (reverse); AMBRA1:5′-TTCCTTTGATGTCTG GGCGT-3′ (forward) and 5′-GGTCCTTGTCAGCTGTCCTC-3′ (reverse); Nrf2: 5′-GCCCTCAGCAT GATGGACTT-3′ (forward) and 5′-GTTTGGGAATGTGGGCAACC-3′ (reverse); p21: 5′-TTCCTTTG ATGTCTGGGCGT-3′ (forward) and 5′-GGTCCTTGTCAGCTGTCCTC-3′ (reverse). qPCR amplifications were carried out on an ABI 7500 Fast-time PCR system (Applied Biosystems, Carlsbad, CA, USA) using SuperReal PreMix Color (SYBR Green) (Tiangen, Beijing, China) and pre-optimized amplification conditions (pre-degeneration at 95 °C for 15 min, denaturing at 95 °C for 10 s, annealing/extending at 60 °C for 30 s). Melting curve data were analyzed to determine PCR specificity. Relative fold expressions were analyzed using the 2^−ΔΔ*C*t^ method and using β-actin *C*_t_ values as the internal reference in each sample.

### 4.10. Transient Gene Silencing by Small Interfering RNAs (siRNAs)

Cells were seeded at a density of 5 × 10^5^ cells/well in six-well plates. Cells were transfected either with siRNA targeting specific genes or scrambled siRNA by means of Lipo6000^TM^ transfection reagent (Beyotime, Shanghai, China) as directed by the manufacturer. The transfection efficiency was measured by Western blot analysis. The siRNA duplexes were synthesized and sequences for the siRNA were as follows: Nrf2 siRNA: 5’-AAUUCCAAGUCCAUCAUGCUG-3’(sense), 5’-GCAUGAUGGACUUGGAAUUGC-3’(antisense), p62siRNA 5’-UAUCAGUUGUACUAAUCCCUU-3’(sense), 5’-GGGAUUAGUACAACUGAUAGU-3’ (antisense).

### 4.11. Statistical Analysis

Statistical analysis was performed using GraphPad prism 5.0 statistical software (San Diego, CA, USA). All of the experiments were performed in triplicate. Data are expressed as mean ± standard deviation. Statistical analysis was carried out using one-way ANOVA, with *p* < 0.05 considered statistically significant.
